# On the cardiorespiratory coordination assessed by the photoplethysmography imaging technique

**DOI:** 10.1038/s41598-023-41828-5

**Published:** 2023-09-05

**Authors:** Stefan Borik, Micha Keller, Volker Perlitz, Simon Lyra, Holger Pelz, Gero Müller, Steffen Leonhardt, Vladimir Blazek

**Affiliations:** 1 Department of Electromagnetic and Biomedical Engineering, Faculty of Electrical Engineering and Information Technology, University of Zilina, Zilina, Slovakia; 2 Department of Psychiatry, Psychotherapy and Psychosomatics, Medical School, RWTH Aachen University, Aachen, Germany; 3 Simplana GmbH, Aachen, Germany; 4 Medical Information Technology (MedIT), Helmholtz-Institute for Biomedical Engineering, RWTH Aachen University, Aachen, Germany; 5 Deutsche Gesellschaft für Osteopathische Medizin (DGOM), Mannheim, Germany; 6 Simplana GmbH, Aachen, Germany; 7 The Czech Institute of Informatics, Robotics and Cybernetics (CIIRC), Czech Technical University in Prague, Prague, Czech Republic

**Keywords:** Biomedical engineering, Imaging and sensing, Circulation, Respiration

## Abstract

Cardiorespiratory coordination (CRC) probes the interaction between cardiac and respiratory oscillators in which cardiac and respiratory activity are synchronized, with individual heartbeats occurring at approximately the same temporal positions during several breathing cycles. An increase of CRC has previously been related to pathological stressful states. We studied CRC employing coordigrams computed from non-contact photoplethysmography imaging (PPGI) and respiratory data using the optical flow method. In a blocked study design, we applied the cold pressure test (CPT), water at ambient temperature (AWT), and intermittent resting conditions. In controls (no intervention), CRC remained on initial low levels throughout measurements. In the experimental group (AWT and CPT intervention), CRC decreased during AWT and CPT. Following both interventions, CRC increased significantly, with a rebound effect following AWT. In controls, HR increased steadily over time. CPT evoked a significant HR increase which correlated with subjective stress/pain ratings. The CRC increase following AWT correlated significantly with subjective pain (*r* = .79) and stress (*r* = .63) ratings. Furthermore, we observed a significant correlation (*r* = − .80) between mean RMSSD and mean duration of CRC, which further supports an association between autonomic state and CRC level. CRC analysis obtained from cutaneous tissue perfusion data therefore appears to be a sensitive and useful method for the study of CRC and ANS activity. Future studies need to investigate the physiological principles and clinical significance of these findings.

## Introduction

Dynamic interactions precipitate in modulations of frequencies and amplitudes of autonomic nervous system (ANS) activity in physiological oscillators such as the cardiac or respiratory systems. A physiological example is e.g. respiratory sinus arrhythmia (RSA). Pathological alterations in frequencies and amplitudes play a pivotal role in medical practice, as exemplified by tachycardia, bradycardia, tachypnea or bradypnea being relevant examples for impaired frequencies, just as arterial hypertension or hypotension denote pathological manifestations in amplitudes. In contrast, phases received considerably less attention in clinical practice though they were scientifically studied more than a 100 years ago^1^. A comprehensive overlook of the manifold systemic physiological functions of relative coordination as a manifestation of phases in explicit examples in animals and humans presented 40 years later also failed to establish phase assessment in medical practice^2^.Introduction of automated simplified assessment of cardio-respiratory coordination using phase positions another 40 years later echoed only poorly in practical medicine^3^. Failure to resonate may have been due to a lack of understanding of the unwieldy term “*phase*” itself and associated clinical ramifications. To an important part, however, this may relate to the fact that none of the manifold analytical tools available for assessing cardiorespiratory coupling (e.g., Granger causality, nonlinear prediction, entropy, symbolization, phase synchronization; see^4^) have yet demonstrated to be useful as a standard clinical application.

Lately, however, two methodological approaches advanced that produced promising insights into the physiology of synchronisation and coordination of phases. In general, coordination or synchronization of cardiorespiratory activity occurs when individual heartbeats are at specific and relatively unchanging temporal positions within successive respiratory cycles. Respiratory cycles represented in the time-domain are referred toas coordination, and, usually, individual heartbeats are mapped relative to the peak of inspiration within a single respiratory cycle. The result of this analysis is termed ‘coordigram’. If respiratory cycles are represented in the phase-domain and, thus, are being normalized to the interval 0 to 2π, the result will be a ‘synchrogram’, and will, thus, depict cardiorespiratory synchronization^5^.

“Synchrograms” on the one side substantially furthered the study of cardiorespiratory phase synchronization (CRS), allowing the observation of the dynamics of cardiac and respiratory rhythms as two weakly coupled but self-sustaining oscillators^5–8^. Increased CRS as an adaptive physiological process has, e.g., been found in healthy (non-)athletes^9^, during meditation^10–12^, and during positive emotional states^13^. “Coordigrams” on the other side probes and complements cardiorespiratory coordination (CRC) as a time-domain measure. Significantly increased CRC during and following pathological apneic sleep states^14^, and increased CRC was also linked to pathological sympathicotonic states of autonomic hyperarousal in preeclamptic women (Berg et al., 2017). More recently, CRC has been demonstrated to be significantly sensitive for the differentiation of workload accumulation when determined by means of principal component analysis (PCA)^15^.

CRC and CRS need to be strictly kept apart since the former is designed to assess coordination by triggering in both directions (e.g. from heart to respiration, and vice versa) to determine respective onsets, whereas the latter determines “a type of cardiorespiratory coupling that manifests through a predilection for heart beats to occur at specific points relative to the phase of the respiratory cycle”^5^ modulated by autonomic activity. In contrast to CRS, CRC captures heart beats fluctuating in time within a given respiratory cycle^5,14^. Methods probing traditionally RSA, such as the frequency analysis, fail to quantify CRC. While the synchrogram examines the heartbeat in relation to respiratory phases, the coordigram associates each heartbeat with the time differences to the preceding and following respiratory onset^5^, thereby rendering the CRC independent of phase computations. This is an important difference as these methods, while very similar, are not identical. The representation of the coordigram allows thus intuitive comprehension of often volatile states of relative coordination. Thereby, while the coordigram is a genuine coupling measure which assesses uni- or bidirectional coordination, it is the behaviour following perturbations which yields better insight into the factual coupling.

Undoubtedly, assessment of cardiorespiratory coordination remains problematic due to the crucial role of two organs involved for the entire organism. Though current technical achievements supply advanced methods for obtaining ECG data and data on respiratory activity than 10 years ago, such new technologies are still somewhat laborious, and it would be desirable to further minimize such efforts. Therefore, the signal of photoplethysmography (PPG) is a reliable, valid, and easy to come by source for cardiorespiratory information. Corresponding investigations demonstrated a high correlation between PPG-derived pulse rate variability and ECG-derived heart rate variability at rest and during mental stress (Stroop Color Test;^16^). This finding was a meaningful achievement since the required PPG-technology is well studied, comparably inexpensive, and can be used at many different sites on the body. Yet another relevant technical advance in PPG technology is given with the development of non-contact, i.e., completely non-invasive PPG imaging (PPGI) systems^17,18^. The application of this new technique in connection with analyses of synchronization and coordination derived from such data would be clinically and practically of utmost advantage since in almost all medical disciplines exact knowledge of ANS activity might be crucial in the diagnosis and therapy of organ actions and interactions.

In a previous communication, we reported on a novel method for evaluating phase shifts in low-frequency (LF) and intermediate (IM) frequency bands employing forehead cutaneous PPGI^19^. In the present study, we employed the same data to investigate the feasibility of non-invasive, non-contact PPGI for determining cardiorespiratory activity as the base of cardiorespiratory coordination. Cardiac activity was detected using PPGI whereas respiratory activity was extracted using the optical flow method. To the best of our knowledge, these imaging methods and signal processing in conjunction with cardiorespiratory coordination analysis have not previously been probed. The primary goal of this study was, therefore, to show the feasibility as well as the efficacy of determining cardiorespiratory coordination solely from non-contact sensing of vital parameters in response to different levels of autonomic load (cold pressure test, CPT; and ambient water test, AWT). This is of sound relevance since there are reports on unexpected increases of coordination in and following severe pathological conditions with autonomic hyperarousal in sleep apnoea^14^ or preeclampsia^20^. Based on these previous findings we hypothesized that ambient/cold pressure test administration would lead to an increase of coordination in subsequent resting conditions. Furthermore, we expected that the effect of the cold and ambient pressure tests on sympatho-vagal interaction from parasympathetic dominance to more sympathicotonic activity be associated with changes in cardiorespiratory coordination.

## Materials and methods

The current study is an extension of a previous study. Therefore, description of the experimental protocol, the methodological setup and the input imaging data are almost identical. All details on the experimental setup and the recording procedure are given in our previous publication on spatial phase distributions^19^.

### Experimental protocol and participants

**Figure 1:**
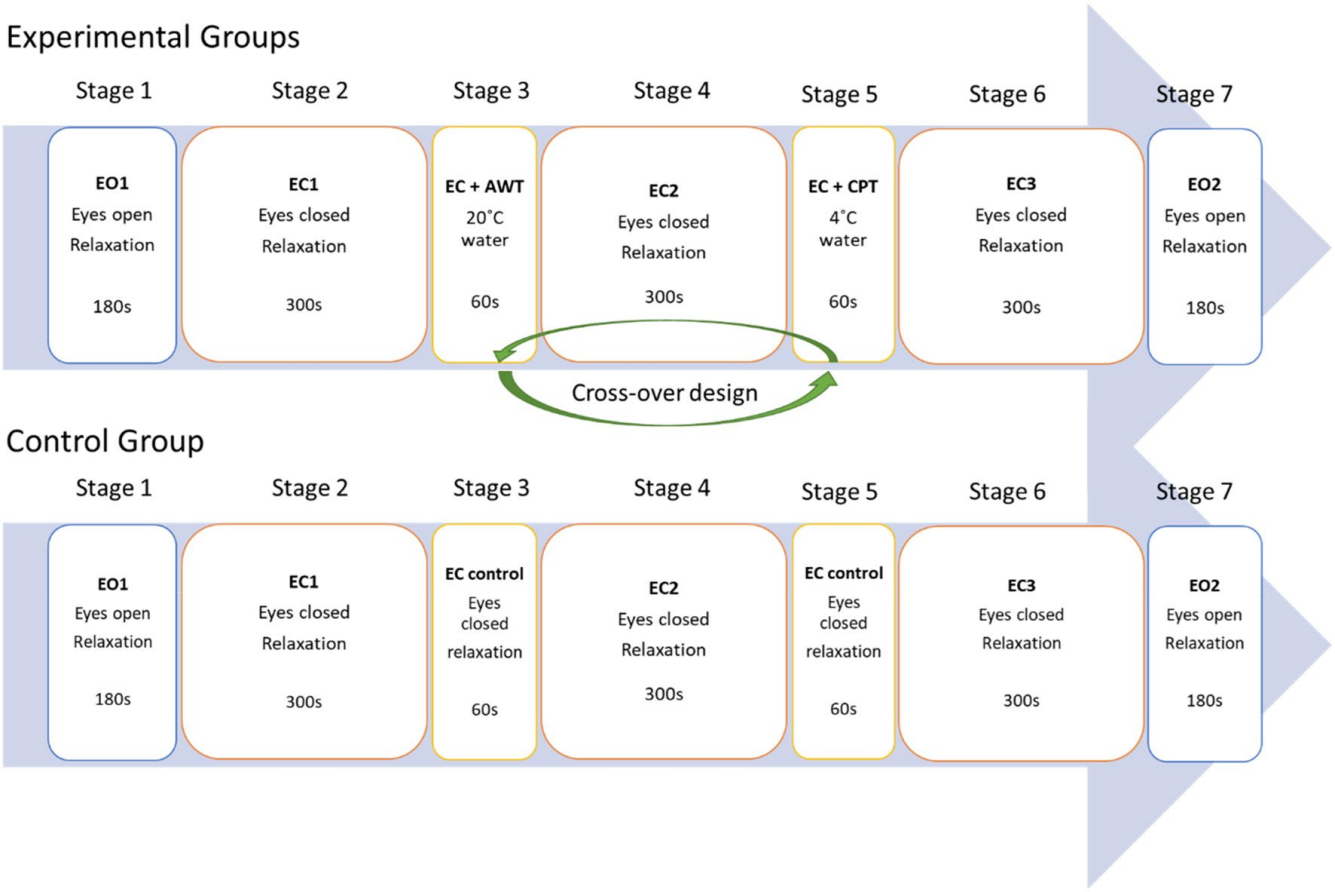
Experimental protocol. Stages of the experiment with (top) warm/cold water test applied in randomized fashion in the experimental group (*N* = 13) and (bottom) no intervention in the control group (*N* = 3).

In short, a total of 16 healthy (BMI < 25; 3 female) non-smoking subjects aged 27.0 (± 2.2) years participated in the experiment performed in supine position. All right- or left-handed individuals were medically checked and had no acute physical or mental illness, as was tested using the German version of the patient health questionnaire (PHQ-D;^21^). The experimental procedure (Fig. 1) consisted of 7 stages: (1) Eyes Open (EO), (2) Eyes closed (EC1), (3) EC + cold pressor/ambient water test (CPT, AWT), (4) EC2, (5) EC + cold pressor/ambient water test, (6) EC3, (7) EO. Stage durations varied (Stage 1/7: 180 s; stage 2/4/6: 300 s; stage 3/5: 60 s). Stage 3 or 5, CPT or AWT, were performed by immersion of the subjects’ non-dominant hand (up to the carpus) in water of either 4 °C or water of ambient temperature (AWT) of 20 °C for 60 s. CPT was used as a sympatho-excitatory manoeuvre to study cardiovascular reactivity^22^, whereas AWT was not expected to cause a sympathetic ANS response. Eyes open and eyes closed served as internal control conditions, revealing the effects of CPT and AWT interventions. The order of CPT and AWT interventions was randomized between subjects in the experimental group. Ten participants received the interventions in the order AWT and CPT (experimental group 1) while this order was reversed in three subjects (experimental group 2). A time control group tested effects of the experimental time length in three subjects without CPT or AWT intervention. During stages 2 to 6, these individuals relaxed in their naïve style keeping their eyes closed. Breathing was not controlled to avoid any external control. Individuals could move their head freely since fixing the head in an attempt to minimize motion artefacts could also increase the experimental load by inducing stress. The entire experiment lasted 23 min in total and was well tolerated by all participants. The study protocol was approved by the ethical committee of the University Hospital at RWTH Aachen University (Ref. No. EK 219–21). Informed consent was obtained from all subjects prior to the study, all methods were performed in accordance with the study protocol and with the Declaration of Helsinki.

### Measurement setup

**Figure 2:**
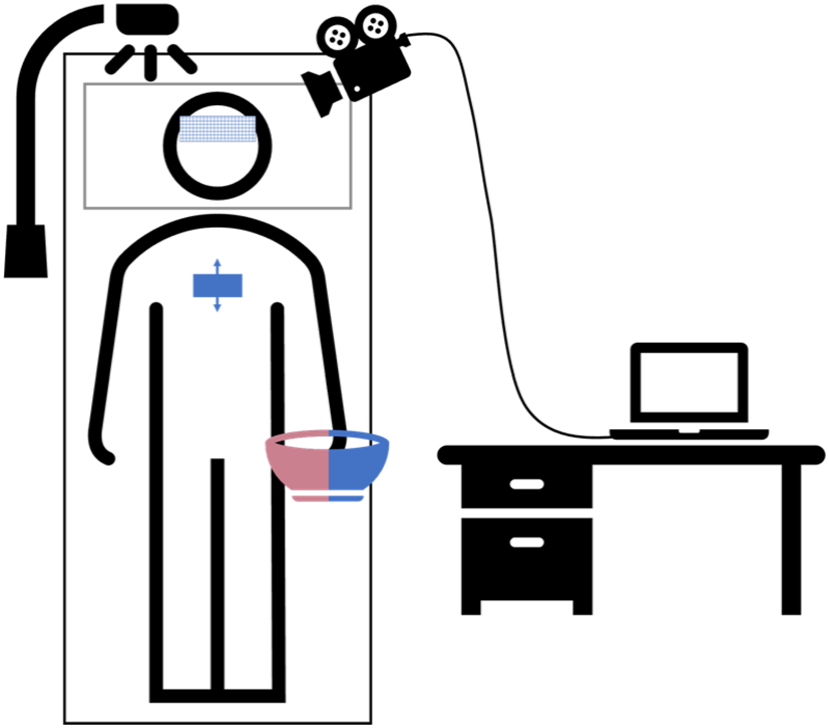
Measurement setup. Subjects lied comfortably on a patient bench. Face and upper part of the were illuminated by a white OLED panel. Regions of interest (ROIs) sensed by the vision camera were the forehead (squared blue ROI) and the chest (solid blue ROI). The examiner assisted subjects to place their hands in bowls with ambient or cold water.

All physiological signals were recorded with a monochrome vision camera, type Grasshopper 3 GS3-U3-23S6 M-C (FLIR Systems, USA). A fixed focal length lens of type Fujinon CF12.5HA-1 (Fujifilm Holdings, Japan) mounted to the camera. The camera was set to sampling rate of 30 frames per second with the frame size set to 1920 × 1200, 12-bit resolution and uncompressed raw format. Regions of interest (Fig. 2) were illuminated using four organic LED (OLED) panels, type Keuka warmwhite 24 V (OLEDWorks, Germany). During the measurement, subjects were lying in a relaxed position on a comfortable patient bench. Optimal conditions for continuous facial skin perfusion measurements were achieved by adjusting the angle of the participant’s head with a pillow. The regions of interest (ROIs) were the forehead and chest. For AWT and CPT, two bowls filled (1) with iced water at 4 °C and (2) water at 20 °C ambient temperature were placed within the subject’s reach to minimize any bodily movements. The entire measurement setup is shown in Fig. 2.

### Signal pre-processing and detection of the cardiac activity

The pre-processing of the signal of perfusion changes in the frontal region was similar as in our previous publication^19^. Here we used *MATLAB* (The MathWorks, Inc., Natick, MA, USA, version R2021a/R2022a). The face was first detected using the Viola-Jones algorithm allowing to search for the subject's face in each frame of the record. This was followed by tracking the frontal ROI using a Kanade-Lucas-Tomasi (KLT) detector. Heart activity related signals (PPGI) were obtained by averaging the entire forehead area. A stationary wavelet transform then detrended the signal and removed motion artefacts. PPGI signal pre-processing was completed by filtering with a bandpass finite response (FIR) equiripple filter (sampling frequency *f*_s_ = 30 Hz, stopband frequencies *f*_stop,1_ = 0.4 Hz and *f*_stop,2_ = 8 Hz, attenuation = 60 dB, passband =  < 0.6–5 > Hz, filter order = 310).

### Signal pre-processing and detection of the respiratory activity

Examination of respiratory data was not within the scope of our previous publication (Borik et al., 2022), but is therefore presented for the first time in the current publication. Following evaluation of different methods to examine respiratory signals, we opted for the optical flow method for supplying optimal properties. Methods such as the direct extraction of the respiratory waveform from the tissue perfusion in the unfiltered PPGI signal may also contain low-frequency oscillations associated with the activity of the ANS (especially in the frontal region, see^23^), an unwanted signal in this case. The optical flow method^24^ extracts movements related to respiration in the thoracic region.

**Figure 3:**
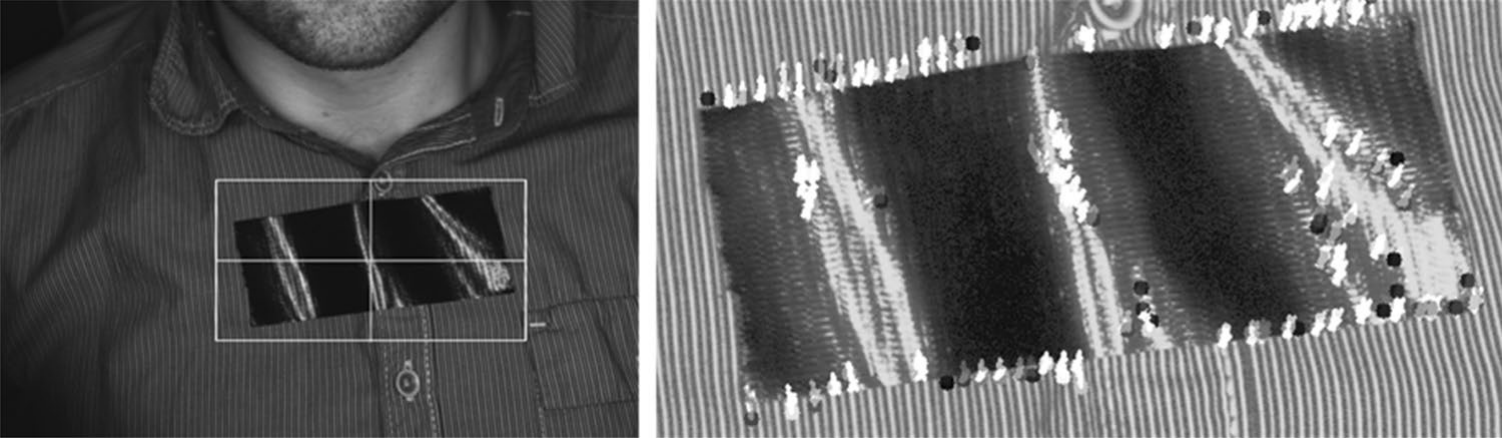
Optical flow. ROI depicted on the left side and tracked motion on the right. Sampling frequency: 5 Hz. Here, showing the record and estimated respiration during 30 s. Black foil glued on the participant’s chest within the ROI to augment contrast.

For the detection of thoracic movements, we used *Python v3.9.6* in combination with the *OpenCV v4.6.0*, *NumPy*^25^ and *Matplotlib*^26^ packages. This method of detecting respiratory activity is implemented by selecting the ROI on the subject's chest used during the experiment (see Fig. 3). A black foil glued on the participant’s chest within the ROI augmented the contrast (Fig. 3). Subsequently, histograms were equalized at this location to reveal details. Then, the KLT algorithm was applied to track movement of the image points corresponding to the respiratory movements of the subject. The detection of respiratory movements was optimized by preserving all details and satisfying the sampling theorem at a sampling rate of 5 Hz. Subsequently, the *MATLAB*-processed signal was oversampled to achieve consistency with PPGI signals sampled at 30 Hz. A stationary wavelet transform was then applied to the respiration signal to remove motion artefacts. Detrending was followed by band-pass filtering using a pair of low-pass and high-pass filters (high-pass filter: FIR, equiripple: *f*_s_ = 30 Hz, *f*_stop_ = 0.01 Hz, attenuation = 60 dB, *f*_pass_ = 0.05 Hz; order = 913; low-pass filter: FIR, equiripple, *f*_s_ = 30 Hz, *f*_stop_ = 0.5 Hz, attenuation = 60 dB, *f*_pass_ = 0.4 Hz; order = 594).

### Creation of coordigrams

**Figure 4:**
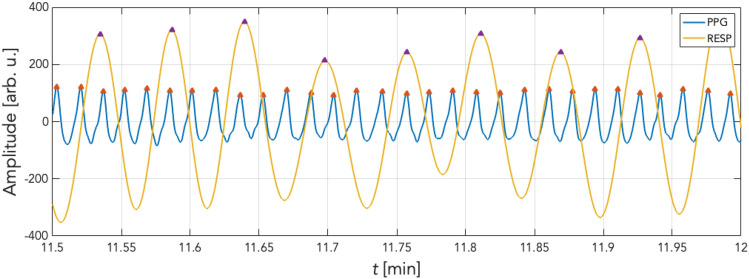
Peak detection. An example showing thirty seconds (11′ 30″ to 12′ at stage 4) of filtered PPG (blue) and filtered respiration (yellow) recordings. Peaks were detected using the *findpeaks*() function (red and violet triangles correspond to detected peaks in each signal).

To assess the coordination and the coupling of cardiac and respiratory activity in individual cycles, the stroboscopic method appeared the most appropriate tool^27^. The preprocessing of PPGI and respiratory signals was followed by detection of local outliers applying a script implemented in MATLAB using the *findpeaks*() function. Figure 4 shows an example of preprocessed respiratory and PPGI signals with their respective detected peaks. Of note, the PPGI signal is shown in its basic form in the reflective mode without inversion for medical interpretation.

**Figure 5:**
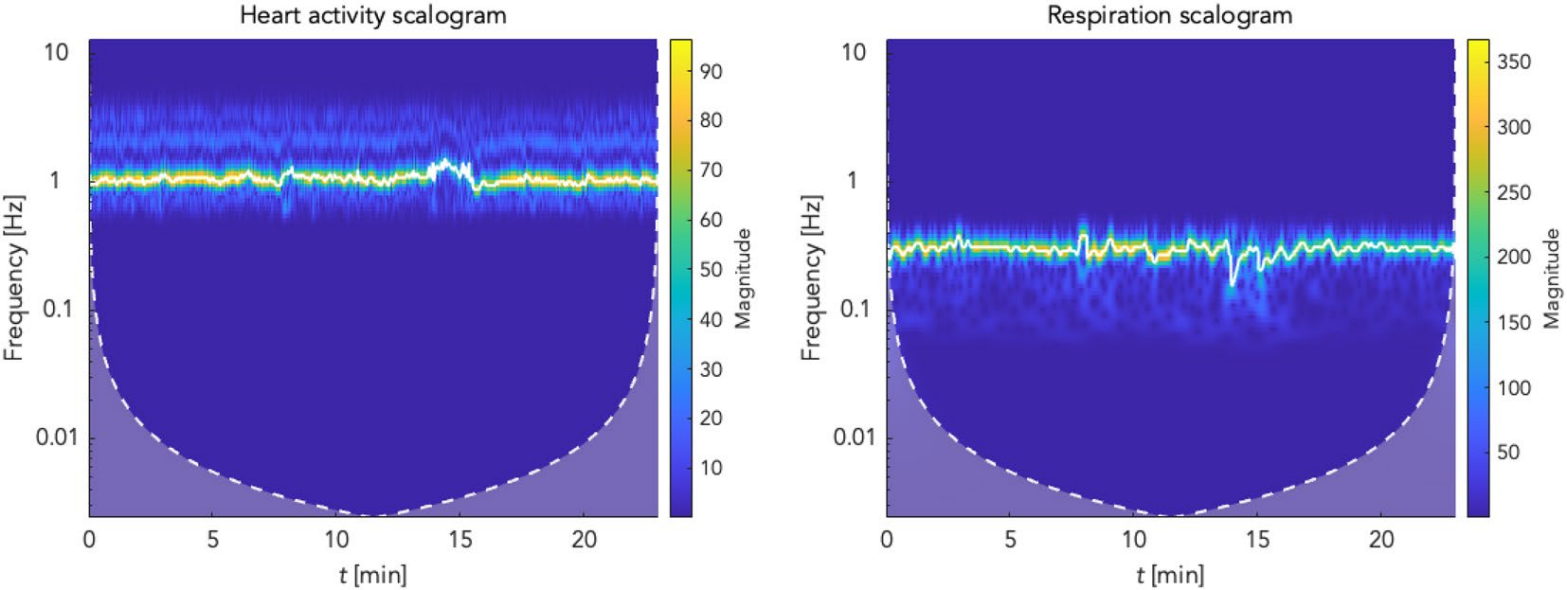
Time–frequency ridges in cardiac and respiratory activity scalograms. The cones of influence (white dashed line) show the time–frequency ridges (white curve) used for estimating the *m*:*n* ratio and optimising the coordination function. The *m*:*n* ratio is the ratio between the instantaneous frequency of heart activity (*m*) and respiratory frequency (*n*), and is used to optimise the coordination function. In selected scalograms, an increase in heart rate can be noticed during the CPT (14 to 15 min of the experiment). It is also possible to observe the change in respiratory rate just during the CPT phase.

**Figure 6:**
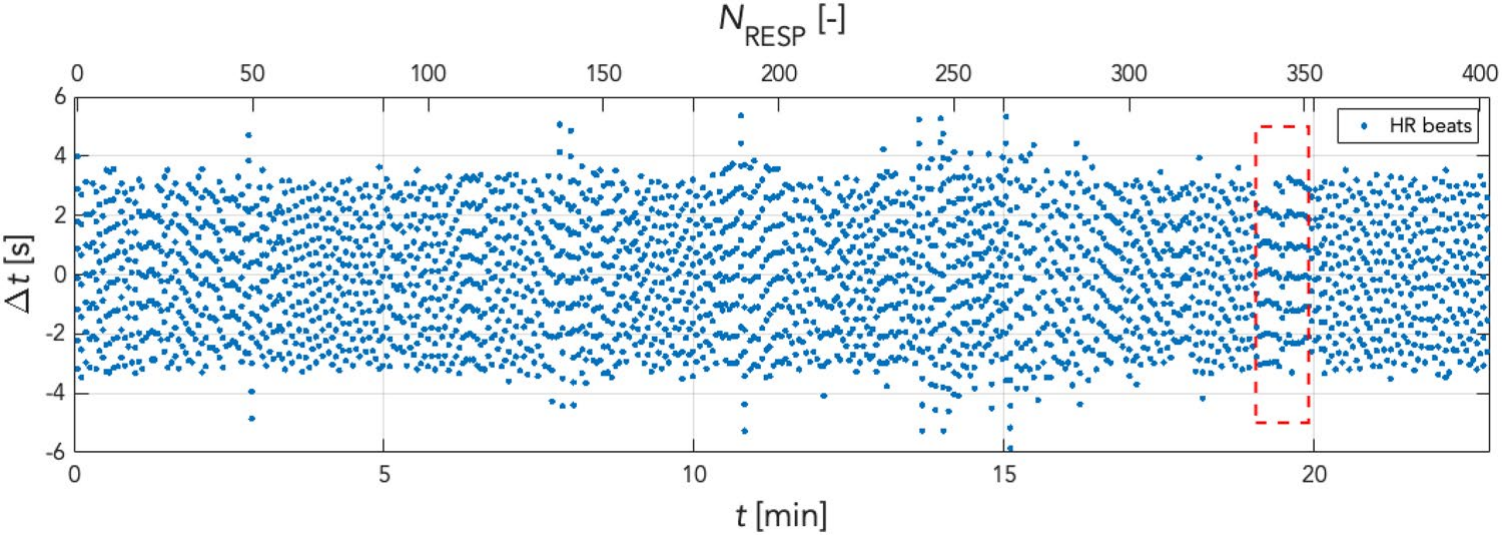
Raw coordigram. Detected heart beats for each respiratory cycle are shown. X-axis: time *t* in min and respiratory cycle numbers *N*_RESP_, Y-axis: The temporal position $\Delta t$ of individual heartbeats relative to the maximum of the corresponding respiratory cycle. The blue dots indicating the individual heartbeats within each respiratory cycle. Heartbeats (blue dots) are always shown in a single vertical line representing one respiratory cycle, with the position. $\Delta t=0 \mathrm{s}$ corresponding to the maximum peak during inspiration. It means that the values $\Delta t<0 \mathrm{s}$ are the positions of the heartbeats during inspiration and $\Delta t>0 \mathrm{s}$ are the temporal positions of the heartbeats during expiration within a single respiratory cycle. The marked box (red dashed line) in the graph shows an episode of horizontal alignment of heartbeats in several consecutive respiratory cycles, the so-called coordination of the two oscillators.

The effect of preprocessing of respiratory and cardiac activity signals can also be observed in the frequency domain. Figure 5 shows the respective scalograms obtained by using the continuous wavelet transform (cwt). In addition to the scalograms, the white line highlights the frequency versus time waveform in the plots computed with the frequency-ridge method. These frequency ridges are later used to optimise the coordination function.After determining the onsets (temporal positions of peaks) in the respiratory and PPGI signals, the raw coordigram (see Fig. 6) can be created by plotting all respiratory onsets/cycles over time by showing the onsets of the individual heartbeats (peaks in the PPGI signal). Figure 6 gives an example showing an increased coordination within a section highlighted by a red dashed line. Here, horizontal lines are formed by heartbeats at approximately the same position as several consecutive respiratory cycles. This phenomenon is referred to as coordination of these two physiological oscillators. Several other similar episodes can be observed in this coordigram. For better interpretability the raw coordigrams were converted into 3D plots where the third dimension is represented by color-coding.

Color-coded coordigrams have been extensively described in the literature^5,14,20^. However, in the following a short summary of our procedure is given. In a moving window containing three breath cycles, the Gaussian function in its parametric form is computed as:

1 $$f\left( {x_{k} } \right) = \frac{1}{{\sqrt {2{\uppi }} }}e^{{ - x_{k}^{2} }} ,$$

while

2 $$x_{k} = \frac{{{\Delta }t_{i} - { }N_{{{\text{HR}},k}} { }}}{b} , k \in 1,N_{{{\text{HR}}}} ,$$

where ${\Delta }t_{i}$ represents the interval duration range of ± 7 s with steps of 0.1 s. This is the interval which was used to compute the cardiorespiratory coordination. At selected intervals and steps, $i \in 1,141$ was used for ${\Delta }t_{i}$. Parameter $N_{{{\text{HR}},{ }k}}$ represents the number of the *k*-th heart cycle, which is gradually incrementing in a moving window for each value of ${\Delta }t_{i}$ in our ± 7 s interval. The moving window size spans three respiratory cycles $N_{{{\text{RESP}}}} = 3$. The *N*_HR_ is the number of the heart cycles in the corresponding moving window. The *b* coefficient equals 0.2 s, similar to^14^, and it represents the sampling time when extracting respiration using the optical flow method. We used this value to avoid blurring the coordigrams in the later detection of coordination episodes.

For each value of $\Delta t_{i}$, the sum is then computed, yielding the matrix ${\mathbf{A}}_{i,r}$:

3 $${\mathbf{A}}_{i,r} = \mathop \sum \limits_{k = 1}^{N} f\left( {x_{k} } \right) ,$$

from which the weighted sum over dimension *r* was calculated:

4 $${\mathbf{C}}_{i,r} = \frac{{2{\uppi }}}{{N_{{{\text{RESP}}}} }}\mathop \sum \limits_{r = 1}^{{N_{{{\text{RESP}}}} }} {\mathbf{A}}_{i,r} ,$$

which yields the matrix **C** of the transformed coordigram, which can be color-coded. Rows represent the temporal position of each heartbeat relative to the peak/centre (maximum of inspiration) of the corresponding respiratory cycle. Thus, negative values on the y-axis represent the heartbeat's positions between the preceding expiration and inspiration, and positive values correspond to values between maximal inspiration and the corresponding expiration. The degree of horizontal alignment is expressed by color, with blue indicating uncoordinated physiological oscillators and yellow a high degree of coordination.

A sample of such a colour-coded coordigram with marked experimental stages is shown in 7, an excerpt from the same recording as given already in 6. Sections with high cardiorespiratory coordination are plotted yellow (close to 1). These color-coded coordigrams allow intuitive visual examination of coordination of two physiological oscillators over time on an individual level. However, automated quantification of coordination for all participants’ coordigrams were transformed to 1D signals. This required introduction of a coordination function created by transforming 3D coordigrams with all columns (and thus all respiratory cycles) into a one-dimensional vector:

1. Use *findpeaks*() to find the local extrema for each column of the coordigram and hence for each respiratory cycle. The minimum peak height (this corresponds to the corresponding heartbeat and its position within the respiratory cycle) is set to 0.75.
2. The quality of this detection was checked using time–frequency ridges of cardiac and respiratory activity (see Fig. 5) comparing the *m*:*n* ratio between instantaneous heart rate (*m*) and respiration (*n*) for each respiratory cycle on the premise that the number of peaks detected for each respiratory cycle must not exceed 2 × (*m*:*n* − 1). If this condition is not satisfied, the coordination function takes the value zero for these columns.
3. The coordination function thus created is then divided by the *m:n* ratio obtained from the time–frequency ridge method from the cwt scalograms. This process ensures values in the approximate range of 0 to 1.

**Figure 7:**
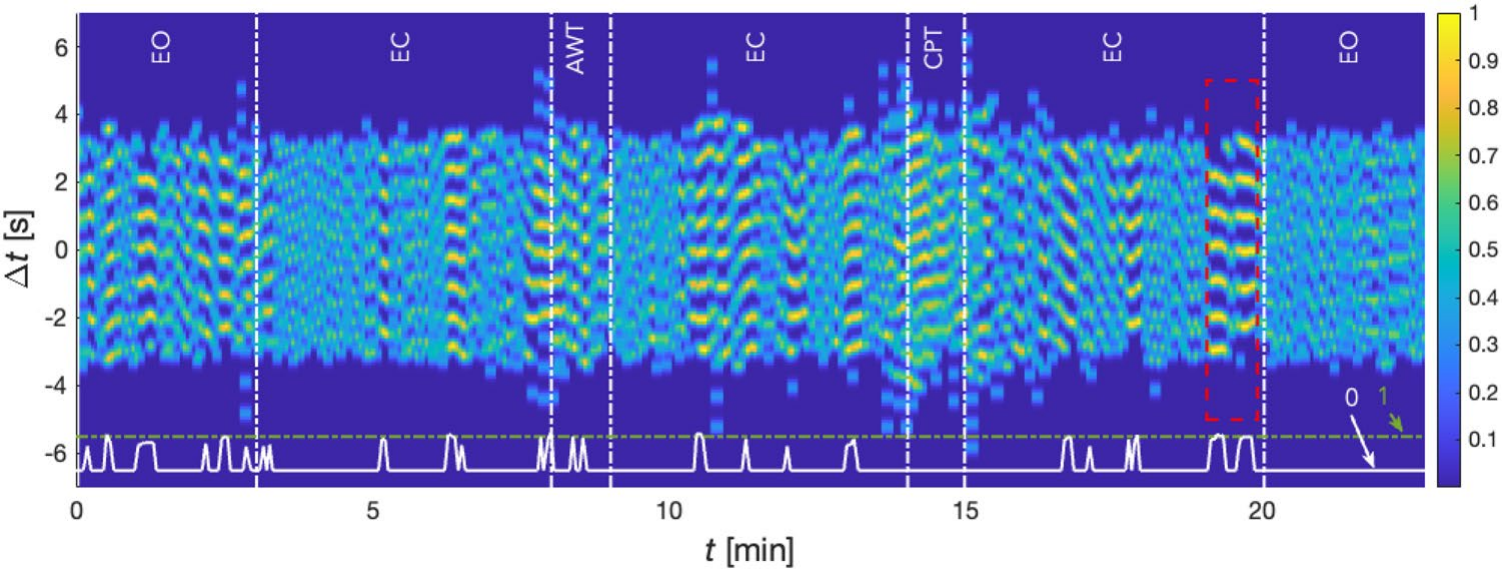
Color-coded coordigram. The degree of coordination of cardiorespiratory activity is color-coded from blue to yellow. Sections of the experiment marked with vertical white dashed lines and labelled on top of the graph. Red dashed lines indicate an example of a section with high degree of cardiorespiratory coordination (same as in Fig. 6). The white line (bottom) represents the time course of the coordination function, which approaches a value of approximately 1 in sections with enhanced coordination (>0.75), and which approaches zero in sections of uncoordinated oscillators. The coordination function allows us to transform the coordigrams into a time course (white line) and thereby map the degree of relative coordination between cardiac and respiratory activity. Thus, the white line depicts a typical scenario where there is temporal coordination that gradually disintegrates and reappears.

The result of this process is given in Fig. 7, where the white line in the bottom of the graph shows the coordination function waveform. During episodes of cardiorespiratory coordination, its value increases. During absence of coordination the value equals zero.

### Statistical analyses

To increase statistical power, experimental groups 1 and 2 were investigated together while the order of CPT and AWT in the second experimental group was changed according to the order within the first experimental group (stages 3,4 <—> 5,6). One participant from the first experimental group was excluded from statistical analyses due to the coordigram being corrupted by movement artefacts. Despite a lack of power in the control group, statistical tests were also conducted for these three participants. According to the main hypothesis it was tested whether the normalized duration of cardiorespiratory coordination would increase following acute autonomic stress evoked by AWT and CPT administration. Therefore, a one-way three level (EC1, EC2 and EC3) repeated-measures ANOVA tested the change of coordination activity. Follow-up paired samples *t*-tests were conducted. Due to the small sample size and accordingly small power as well as the exploratory nature of analyses, follow-up paired *t*-tests were not corrected for multiple comparisons. A *p*-value of .05 was considered statistically significant.

The root mean square of successive RR interval differences (RMSSD) was computed for cardiac and respiratory data for each experimental stage^28^. *MATLAB* (The MathWorks, Inc., Natick, MA, USA, version R2021a/R2022a) was used to compute RMSSD for 60 s sections across the experiment. For cardiac data, this time range has been shown to provide accurate RMSSD estimates (e.g.,^29^). Average RMSSD values for longer stages were computed by taking the mean of several 60 s sections. In addition, we also followed a similar procedure for the calculation of respiratory RMSSD. To further examine the validity of computed coordination data, the mean coordination across the whole measurement was correlated (Pearson’s *r*) with mean values of cardiac and respiratory RMSSD data.

For further exploratory analyses, responses during AWT and CPT administration were examined. Visual inspection of 3D coordigrams showed heterogeneous coordination activity following AWT and CPT across participants. Furthermore, scalograms suggested that not all participants responded with an increase of HR during CPT. To further investigate the effect of individual responses to AWT and CPT, follow-up explorative analyses were computed. Physiological stress responses during AWT and CPT were investigated by analysing instantaneous heart rate waveforms extracted from scalograms using a time–frequency ridge (TFR) (see Fig. 5). Absolute HR values [bpm] as well as respiratory rates [cpm] are reported. Extracted TFRs for HR were then min–max normalized for investigation on the group level. HR change was computed as the change from the eyes-closed condition to AWT or CPT (‘AWT–EC1’ or ‘CPT–EC2’). In this way, we tried to track a change/increase in HR, which can be considered a stress response.

To further improve the assessment of physiological responses, a questionnaire on subjective psychological experience to CPT administration was mailed to participants 6 months after their participation since this measure was not surveyed at the time of recordings. Participants were asked to rate their memory of experiencing stress or pain during AWT and CPT administration on a scale from 0 (no stress/pain) to 5 (maximal stress/pain). To further test the influence of autonomic stress experience during AWT and CPT administration, two-sided Pearson’s *r* correlations were computed between coordination change (AWT change computed as ‘EC2–AWT’; CPT change computed as ‘EC3–CPT’) and subjective stress and pain experience as well as physiological HR reaction. Here, we assumed that the stress response could also be induced after AWT and CPT, which determined how we computed the coordination changes in this case.

## Results

### Repeated measures ANOVAs of cardiorespiratory coordination

**Figure 8:**
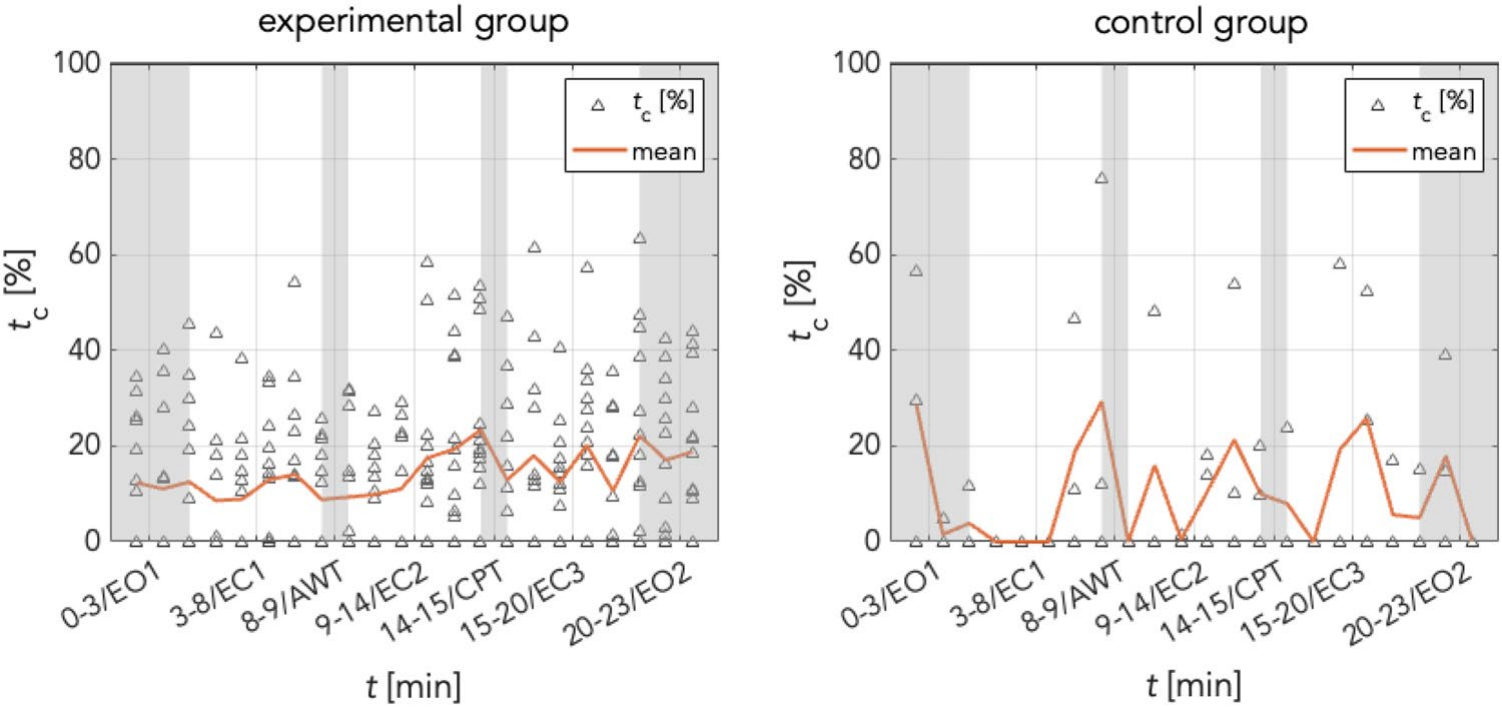
Cardiorespiratory coordination (CRC). Relative ratio in [%] of CRC in (left) experimental group and (right) control group. Whereas the experimental group displays a stable increase from EC1 to EC2/3, the control group only shows random (non-significant) fluctuations. This indicates that change of CRC in the experimental group may indeed be an effect of ambient/cold pressure.

In the experimental (AWT + CPT) group, a one-way repeated measures ANOVA revealed a marginally significant effect of factor condition (EC1, EC2 and EC3) on normalized mean durations of coordination between cardiac and respiratory oscillators (*F*(22,2) = 3.45, *p* = .05; Table 1). The assumption of sphericity was met (*χ*^2^(2) = 3.2, *p* = .2). Post-hoc paired *t*-tests further indicated a significant difference between EC1 and EC2 (*t*(11) = − 2.2, *p* = .05), a significant difference between EC1 and EC3 (*t*(11) = − 3.02, *p* = .01), but no significant difference between EC2 and EC3 (*t*(11) = .29, *p* = .78). Post-hoc *t-*tests were not corrected for multiple comparisons, however, the increase of coordination from EC1 to EC3 would have survived correction. This indicates that CRC increased following the AWT condition, and remained at a stably increased level following CPT (see Fig. 8). However, the CRC increase after AWT and the continued increase following CPT (after initial decrease of CRC during CPT) suggests that both AWT and CPT affected CRC. A one-way repeated measures ANOVA for the control group revealed no significant increase of CRC across EC conditions (*F*(4,2) = .13, *p* = .80). Again, the assumption of sphericity was met (*χ*^2^(2) = .54, *p* = .80). Despite the limited sample size, the stable level of CRC in the control group further suggests that AWT or CPT conditions were both potent to induce changes in CRC.

**Table 1:** Descriptives (mean ± SD) of cardiorespiratory coordination (CRC) activity per experimental condition and repeated measures ANOVA of eyes-closed conditions.

Group	EO1	EC1	AWT	EC2	CPT	EC3	EO2	*F*	*p*
Experimental group	.13 (± .12)	**.12 (± .09)**	.10 (± .13)	**.18 (± .07)**	.14 (± .16)	**.17 (± .07)**	.18 (± .1)	*F*(22,2) = 3.45	***p* = .05**
Control	.11 (± .10)	**.11 (± .12)**	.00	**.12 (± .14)**	.08 (± .14)	**.11 (± .15)**	.10 (± .14)	*F*(4,2) = .13	*p* = .80

Significant values are in bold.

### Validation check of cardiorespiratory coordination

**Figure 9:**
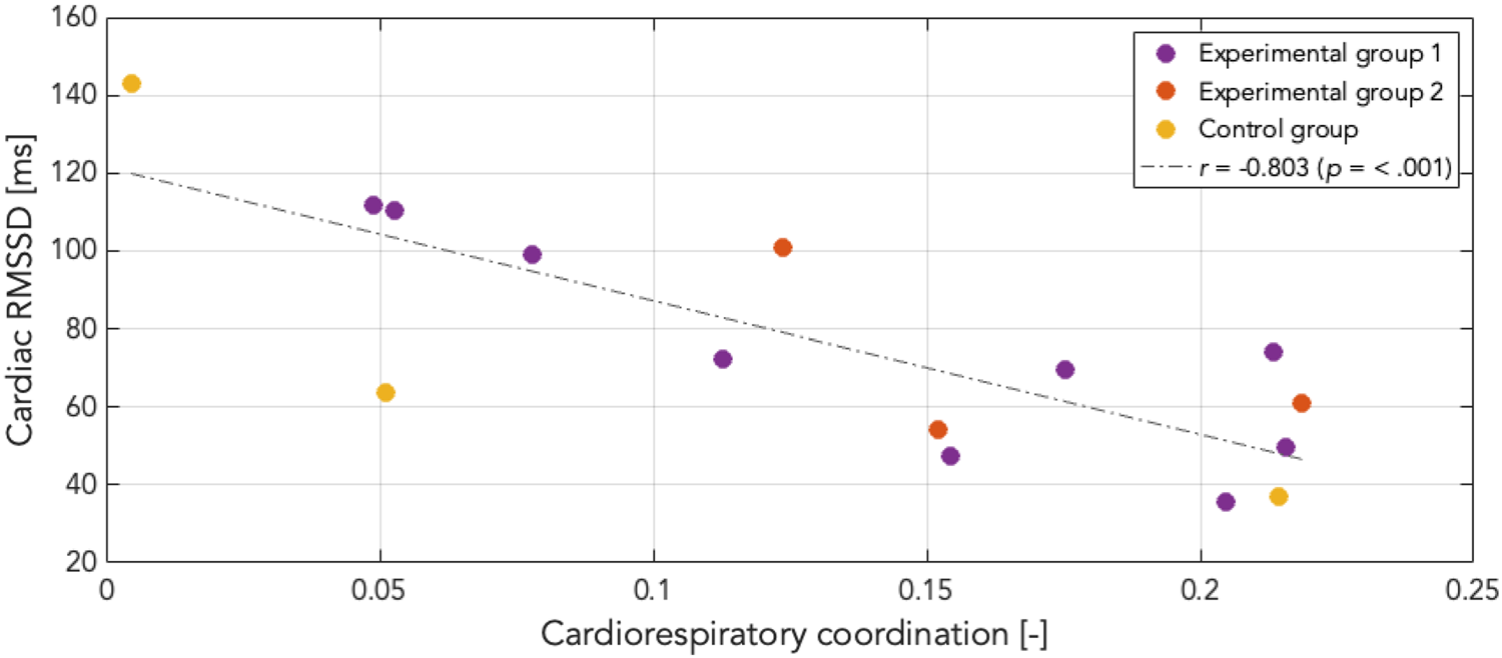
Correlation of cardiac RMSSD and cardiorespiratory coordination (CRC). Negative correlation between mean cardiac RMSSD values and CRC throughout the whole measurement and across all groups. Elevated cardiac RMSSD levels correspond with low CRC levels, and vice versa. Higher CRC during states of sympathetic dominance are in line with increased CRC in pathological states.

To test the validity of observed CRC values, mean RMSSD for cardiac and respiratory data as well as normalized CRC values were computed across the entire measurement, independent of experimental stage. Mean RMSSD values for each experimental stage are reported in Table 2. RMSSD has been shown to be a marker of cardiac vagal control, i.e., a physiological indicator of self-regulation^28,30^. A Pearson’s correlation of *r* = − .80 (*p* < .001) showed a strong negative association between coordination and parasympathetic dominance for cardiac data (Fig. 9), but a non-significant negative association for respiratory data (*r* = − .53, *p* = .08). This suggests that participants with higher cardiac RMSSD values, i.e., more parasympathetic activity and better self-regulation, displayed lower CRC. Increased CRC values in states of sympathetic dominance are in keeping with previous findings which have shown increased CRC in pathological states defined by autonomic hyperactivity, see e.g.,^14^.

**Table 2:** Mean (± SD) heart rate [bpm] and respiratory rate [cpm] across experimental conditions and change from EC1 to AWT and EC2 to CPT, as well as RMSSD [ms] for cardiac and respiratory data.

Variable	Group	EO1	EC1	AWT	EC2	CPT	EC3	EO2	AWT-EC1	CPT-EC2
HR	Experimental	66.6 (± 10.0)	67.1 (± 10.1)	66.0 (± 10.8)	65.9 (± 9.0)	70.8 (± 10.4)	64.8 (± 8.8)	63.4 (± 9.1)	− 1.1 (± 2.8)	4.9 (± 7.6)
	Control	56.5 (± 8.3)	56.8 (± 8.4)	54.1 (± 9.1)	55.3 (± 8.2)	61.3 (± 13.1)	56.4 (± 7.3)	58.5 (± 10.2)	− 2.7 (± .7)	5.9 (± 5.2)
Resp	Experimental	13.6 (± 4.0)	12.9 (± 4.4)	13.6 (± 4.2)	14.7 (± 4.0)	15.1 (± 2.8)	14.2 (± 3.7)	15.0 (± 4.0)	.67 (± 1.7)	.40 (3.0)
	Control	13.3 (± 3.9)	12.9 (± 3.8)	13.4 (± 3.8)	13.5 (± 3.9)	12.7 (± 5.8)	12.7 (± 5.0)	10.6 (± 4.0)	.54 (± 1.0)	− .75 (1.9)
RMSSD cardiac	Experimental	83.6 (± 45.7)	69.2 (± 27.8)	77.5 (± 29.6)	67.2 (± 22.2)	80.0 (± 28.7)	71.0 (± 21.4)	68.8 (± 28.8)	7.9 (± 19.2)	11.7 (± 17.3)
	Control	88.8 (± 71.5)	76.1 (± 59.0)	83.6 (± 69.1)	81.5 (± 38.7)	90.9 (± 59.0)	73.2 (± 45.8)	74.1 (± 49.9)	7.5 (± 10.2)	9.4 (± 21.1)
RMSSD respiratory	Experimental	1.81 (± .60)	1.85 (± .60)	2.24 (± .90)	2.09 (± .60)	2.18 (± .90)	2.15 (± .50)	1.86 (± .60)	.34 (± .9)	.11 (± .7)
	Control	1.53 (± .50)	1.35 (± .30)	1.96 (± .90)	1.98 (± .80)	2.37 (± .10)	2.15 (± .30)	1.70 (± .60)	.61 (± .7)	.40 (± .9)

### Follow-up exploration based on physiological and psychological responses

**Figure 10:**
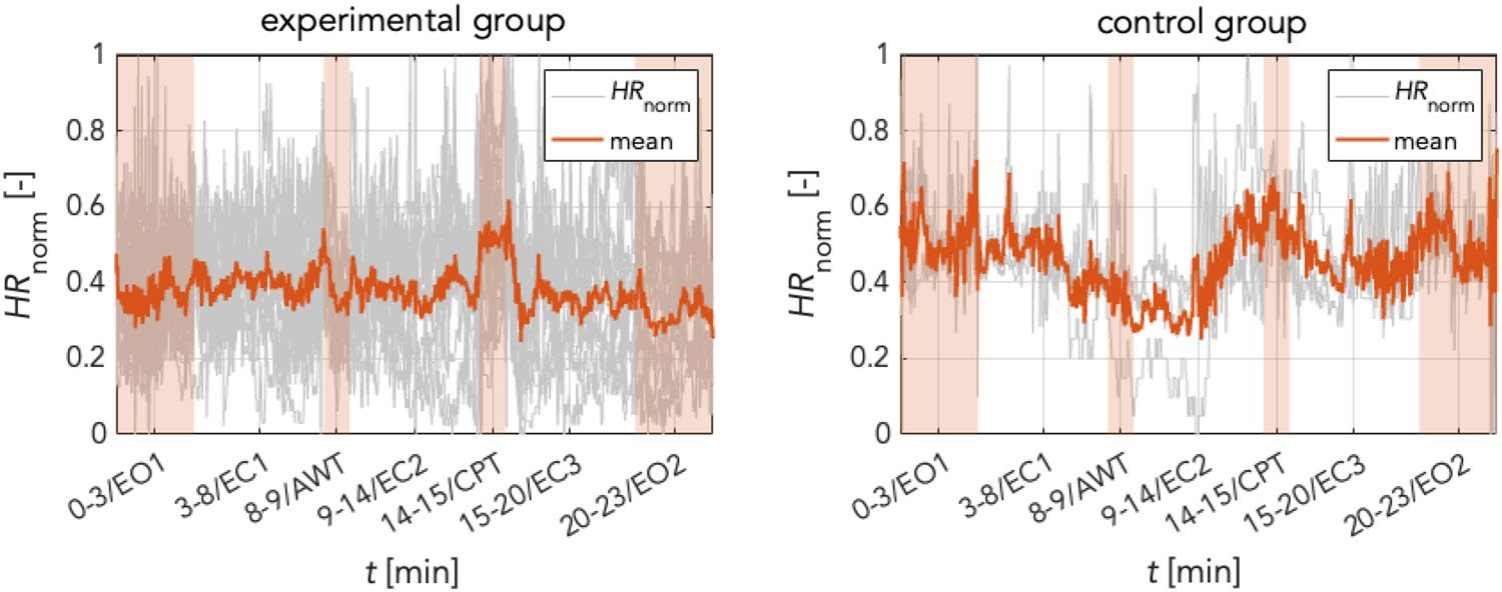
Normalized heart rates (TFRs) for (A) main + random group and (B) control group. X-axis: time [min], Y-axis: grey: time courses for individual HR, red: mean HR time courses; CPT at 14–15 min. Normalization of the heart rate allows us to better compare the physiological responses of the different subjects involved in the experiment. In the case of the experimental group, we can notice oscillations in HR throughout the experiment with a significant increase during the CPT.

Participants’ individual scalograms exhibited individually different physiological responses especially during CPT. Therefore, physiological ANS responses during CPT were computed extracting instantaneous heart rate waveforms from normalized time–frequency ridges (TFRs) obtained from scalograms (see Fig. 5). The normalized heart rate of all groups is depicted in Fig. 10. This showed no significant changes of HR from EC1 to AWT in either group (experimental: mean HR_EC1_ = .39 (± .09), mean HR_AWT_ = .37 (± .08), *t*(11) = 1.12, *p* = .30; controls: mean HR_EC1_ = .45 (± .02), mean HR_AWT_ = .33 (± .13), *t*(2) = 1.6, *p* = .24). However, HR changes were significant from EC2 to CPT in experimental group (mean HR_EC2_ = .37 (± .12), mean HR_CPT_ = .52 (± .16), *t*(11) = 2.3, *p* < .05; Table 2). This suggests that some participants clearly responded to CPT whereas others did not. In controls, HR changes showed a heterogeneous pattern with a non-significant increase from EC2 to CPT (mean HR_EC2_ = .4 (± .05), mean HR_CPT_ = .57 (± .07), *t*(2) = − 2.45, *p* = .13; Table 2). Descriptively, there was a decrease of HR from EC1 to AWT and an increase from EC2 to CPT. Furthermore, to resolve variance in ANS responses during CPT we assessed subjective stress and pain perceptions of a short survey conducted some 6 months after completing biometric recordings, see Table 3.

**Table 3:** Perceived stress and pain ratings during administration of cold and ambient pressure task for the experimental group (N = 13).

CPT		AWT	
Stress (*M* ± *SD*)	Pain (*M* ± *SD*)	Stress (*M* ± *SD*)	Pain (*M* ± *SD*)
2.7 (± 1.1)	3.0 (± 1.2)	1.1 (± 1.2)	1.5 (± 1.4)

Despite the time gap between biometric recording and completing the stress/pain related psychometry, ANS CPT responses (‘HR CPT’–‘HR EC2’) were highly correlated with psychological responses throughout the whole main + random group (stress: *r* = .66, *p* = .02; pain: *r* = .65, *p* = .02). High correlations between objective ANS measures and subjective psychological stress responses supports that participants reliably memorized their physiological experience during the study.

To further test the influence of ANS responses during AWT and CPT, two-sided correlations were computed between CRC changes (AWT change computed as “EC2–AWT”; CPT change computed as “EC3–CPT”), the subjective stress/pain experience, and ANS related HR reactions (see Table 4).

**Table 4:** Pearson’s correlations between CRC changes, subjective and physiological reactions during ambient water task (AWT) and cold pressure task (CPT).

Variable	AWT				Variable	CPT			
	1	2	3	4		1	2	3	4
1. CRC change	–				1. CRC change	–			
	–					–			
2. HR change	− .19	–			2. HR change	.43	–		
	(.56)	–				(.17)	–		
3. AWT stress	**.63**	− .07	–		3. CPT stress	.11	**.66**	–	
	**(.04)**	(.84)	–			(.73)	**(.02)**	–	
4. AWT pain	**.79**	.03	**.87**	–	4. CPT pain	− .07	**.65**	**.70**	–
	**(.004)**	(.93)	**(< .001)**	–		(.83)	**(.02)**	**(.01)**	–

Reported as correlation coefficients r (*p*-value).

Significant values are in bold.

The correlations were not corrected for multiple testing and were computed for exploratory purposes. CRC changes for AWT (changes from AWT to EC2) revealed a positive significant correlation with stress (*r* = .63, *p* = .04) and pain (*r* = .79, *p* = .004) ratings. Higher stress and pain ratings were therefore associated with a higher increase of CRC after AWT. Furthermore, there was a high correlation between stress and pain ratings (*r* = .87, *p* < .001). However, there was no significant correlation between HR changes and CRC or subjective stress and pain ratings for AWT.

For CPT, the subjective stress and pain ratings were significantly correlated with the HR change (stress: *r* = .66, *p* = .02; pain: *r* = .65, *p* = .02), revealing a significant overlap of subjective experiences during CPT and physiological responses. Furthermore, there was a positive significant correlation between stress and pain ratings (*r* = .70, *p* = .01). Interestingly, CRC changes induced by CPT were not significantly correlated with subjective or physiological CPT responses. However, descriptive CRC was already increased following AWT administration, and, therefore, it is possible that further effects are biased by carryover-effects from previous stages.

## Discussion and conclusion

Despite recent investigations on the coordinated interactions in cardiovascular and cardiorespiratory systems, this area of research is still in its infancy. However, several linear and non-linear methods introduced lately to evaluate the interactions in these dynamic systems (for a review, see^4^) bear potentials to advance this field. Ease of applying these tools should accelerate their acceptance in medical research and clinical settings given additional support on their physiological and systemic significance. On these grounds it was the purpose of this study to further the accessibility and use of these technologies by demonstrating computation of cardiorespiratory coordination (CRC) from completely non-invasively recorded skin blood flow data. We detected cardiac activity with photoplethysmography imaging data (PPGI^17^ and we determined respiratory activity from thesee data using an image processing method based on optical flow evaluation analysis^24^. Given these technical approaches, we probed CRC in several randomized conditions, at rest, at an ambient water temperature exposition, and at a cold pressure test (AWT, CPT, resp.). Three participants undergoing only rest conditions served as time control group. Overall, we found a significant negative correlation of mean CRC and mean RMSSD indicating higher parasympathetic influence related to reduced CRC across measurements. This is in line with reports on increased CRC in states of gross autonomic arousal, see e.g.,^14^. A negative correlation of mean CRC and mean RMSSD for respiratory data, however, did not reach significance. Our findings support the general feasibility and physiological validity of CRC computations based on employing non-invasive, non-contact imaging data.

To the best of our knowledge, this is the first study to apply CRC analyses employing the CPT/AWT paradigm. A general sparsity of published research on coordination as well as inconsistent methodologies further complicate the interpretation of our findings. One previous study demonstrated highly significant increases of CRC in light sleep, despite increases in sympathetic activity and metabolic transport due to extended apnoeic periods^14^. This contradicted previous reports on diminished CRC supposedly originating in increasing metabolic transport and a sympathetic dominance of the excitatory branch^3,31^. Additionally, CRC during different sleep phases is comprehensively investigated in^32^. where, the different sleep phases are assessed with the "on" and "off” periods of the cardiorespiratory coupling showing intermittent nature of CRC. In the current study, we observed different courses of CRC and HR in the experimental groups and controls, respectively. While AWT and CPT were followed by increases in CRC in the experimental groups, CRC remained on a constant level in controls showing only spontaneous CRC fluctuations. A tonic level of CRC around 0.1 appears in keeping with reports on increased CRC levels during physical rest conditions^33^. AWT and CPT applied in our experimental groups appeared as effective catalysts for increasing CRC. Surprisingly, AWT evoked a significant increase of CRC during EC2, followed by a slight decline during CPT and a rebound to CRC levels of EC2. Due to the unbalanced design of experimental groups, a carryover-effect from experimental conditions cannot be excluded at this point. For HR, outcomes were in reverse order. HR remained on comparable levels over time in experimental groups while it slowly (yet non-significantly) increased in controls (see Tables 1 and 2). This may signify that relaxation may be experienced as unnerving when lasting beyond 7 min^34^.

Our findings on CRC appear to parallel findings in a previous study using the same data reporting graded slow and constant increases in phase shifts in different frequency bands in the experimental groups^19^. This investigation supported the inclusion of a third, the so-called intermediary (IM) band besides ANS-related low and high frequency (LF and HF) bands when assessing any systemic effects in experiments in humans. The IM band concept rests on findings on peripheral synchronisation in respiration, arterial blood pressure, and skin blood flow associated with decreased levels of reticular activity in the brainstem^35^. This is also in keeping with early reports on IM band emergence following short-term hyperventilation^36^. The IM band concept has been confirmed lately in several fMRI studies^37,38^. Comparable to our findings in previous phase shift analyses^19^, the increase of CRC following AWT as well as CPT may thus be related to a self-regulatory mechanism supported by adaptation of the ANS. Such a concept on systemic self-organization has also been proposed for the activity of the default mode network (DMN) (for a review, see^13^).

An apparent parallel response on the CRC and ANS level is also corroborated by our findings on the inverse relationship between CRC and RMSSD levels with low RMSSD being accompanied by elevated CRC, and vice versa. While the RMSSD measure needs to be viewed with some precaution^39,40^, it is yet to be considered another valid piece contributing eventually to a slightly less puzzling picture.

Other work that is consistent with our findings is focused on the analysis of CRC during cardiorespiratory stress using a principal component analysis (PCA) approach^15^. The authors also found changes in CRC with the accumulation of fatigue (i.e., with a decline in parasympathetic and an increase in sympathetic activity).

Throughout the whole measurement, control participants remained on the same numeric CRC level showing only spontaneously scattered CRC fluctuations. Examination of HR changes from rest condition to AWT or CPT showed that only CPT evoked a significant stress response whereas controls showed marked spontaneous fluctuations, see Table 2. In the experimental groups, CPT was accompanied by an immediate increase in HR, most likely due to a declining vagal tone as an ANS adaptation to CPT. Such physiological responses were to be expected, and were further corroborated by a post-hoc questionnaire probing the individually experienced mental stress and pain during AWT and CPT. Here, CPT stress and pain ratings were positively correlated with HR changes (EC2 to CPT) while there was no such correlation for AWT. Interestingly, however, CRC changes in AWT (changes from AWT to EC2) were positively correlated with AWT stress and pain responses which was not found for CPT. The positive association of stress and pain responses and CRC changes following AWT is an interesting finding, suggesting that subjective experiences, CRC as well as autonomic balance may be intertwined.

The modality used for physiological recordings and analyses presented with this communication allows a widely facilitated yet scientifically sound assessment of CRC. This is of explicit benefit in circumstances where it is impossible to record signals from patients with contact technology, e.g., in new-borns in incubators or persons suffering skin damages such as following severe burn injuries. Thus, we are confident that these tools will facilitate much-needed future clinical investigations, which can even be accomplished with conventional PPG only. Such advances should ultimately help establish the hitherto confounding processes of synchronisation and coordination further in the clinical world.

## Limitations

The current findings present a novel technological approach of investigating cardiorespiratory coordination in PPGI and camera based respiratory data. However, reliability of findings should be further investigated in a larger and more balanced sample as a correlation between coordination and subjective stress/pain ratings was found for AWT but not for CPT. The unbalanced between-subject cross-over design with ten out of thirteen participants receiving the intervention in AWT/CPT order may have impeded findings of significant associations between CRC and subjective or physiological responses during CPT. Secondly, our experimental stages varied in duration which may have impeded comparability between sections. However, robustness to abrupt temporal changes is exactly the stronghold of the CRC approach. Lastly, the non-contact nature of the applied method also has inherent drawbacks such as motion artefacts, which may be laborious or even impossible to eliminate entirely. For clinical use, automatic artefact identification and correction methods need to be advanced further. Despite the limitations of our study, our findings yet support an intricate interaction between ANS and CRC.

## Outlook

Using PPGI and the optical flow method we presented first evidence that recording and computation of CRC are feasible in an easy, valid, and reliable mode. Our results support the current state of research on CRC and pave the way for future studies using this technology such as directionality of cardiorespiratory interactions (see Krause et al. 2017). This non-contact and non-invasive method also enables the recording of physiological signals in patients who are difficult to measure in direct contact technologies (e.g., newborns, patients with severe burns). Future studies should not only further corroborate the reliability of this technique but also investigate the physiological significance of CRC in varying groups of patients in various experimental or even natural environments.

## Data Availability

The datasets used and analysed during the current study are available from the corresponding author on reasonable request.
